# Synthesis and Diels–Alder Reactivity of 4-Fluoro-4-Methyl-4*H*-Pyrazoles

**DOI:** 10.3390/ijms21113964

**Published:** 2020-05-31

**Authors:** Nile S. Abularrage, Brian J. Levandowski, Ronald T. Raines

**Affiliations:** Department of Chemistry, Massachusetts Institute of Technology, Cambridge, MA 02139-4307, USA; nile@mit.edu (N.S.A.); levandbj@mit.edu (B.J.L.)

**Keywords:** antiaromaticity, click chemistry, cycloaddition, 4*H*-pyrazole, hyperconjugation

## Abstract

4*H*-Pyrazoles are emerging scaffolds for “click” chemistry. Late-stage fluorination with Selectfluor^®^ is found to provide a reliable route to 4-fluoro-4-methyl-4*H*-pyrazoles. 4-Fluoro-4-methyl-3,5-diphenyl-4*H*-pyrazole (MFP) manifested 7-fold lower Diels–Alder reactivity than did 4,4-difluoro-3,5-diphenyl-4*H*-pyrazole (DFP), but higher stability in the presence of biological nucleophiles. Calculations indicate that a large decrease in the hyperconjugative antiaromaticity in MFP relative to DFP does not lead to a large loss in Diels–Alder reactivity because the ground-state structure of MFP avoids hyperconjugative antiaromaticity by distorting into an envelope-like conformation like that in the Diels–Alder transition state. This predistortion enhances the reactivity of MFP and offsets the decrease in reactivity from the diminished hyperconjugative antiaromaticity.

## 1. Introduction

Discovered in 1928 [1], the Diels–Alder reaction has become a mainstay of synthetic organic chemistry and allied fields [2,3,4,5,6]. Diels–Alder reactions of 4*H*-pyrazoles, in particular, have been explored by the groups of Adam and Hünig [7,8,9,10]. 4*H*-Pyrazoles with alkyl or chloro substituents at the saturated center require acid catalysis to proceed, whereas 4*H*-pyrazoles bearing electron-withdrawing fluoro substituents react rapidly as Diels–Alder dienes in the absence of an acid [11]. Density functional theory (DFT) calculations show that the uncatalyzed Diels–Alder reaction of 4,4-difluoro-3,5-diphenyl-4*H*-pyrazole (DFP) proceeds ~500,000 times faster than does the reaction of 4,4-dimethyl-3,5-diphenyl-4*H*-pyrazole with the strained alkyne, *endo*-bicyclo[6.1.0]non-4-yn-9-ylmethanol (BCN). This increase in reactivity is the result of the hyperconjugative antiaromaticity [12,13,14,15] and the lowering of the lowest unoccupied molecular orbital (LUMO) energy induced by the electron-withdrawing fluoro substituents.

Our group is interested in developing the 4*H*-pyrazole scaffold as a reagent for “click” chemistry [16]. Recently, we showed that the 4,4-difluoro-4*H*-pyrazole scaffold is more reactive than the tetrazine scaffold towards BCN. The rapid Diels–Alder reactivity came, however, with a reduction in the stability of the diene scaffold toward biological nucleophiles, which limits the utility of 4,4-difluoro-4*H*-pyrazoles in physiological environments. In an effort to maintain rapid Diels–Alder reactivity without sacrificing stability, we have turned our attention to the facially asymmetric 4-fluoro-4-methyl-4*H*-pyrazole scaffold.

**Scheme 1:**
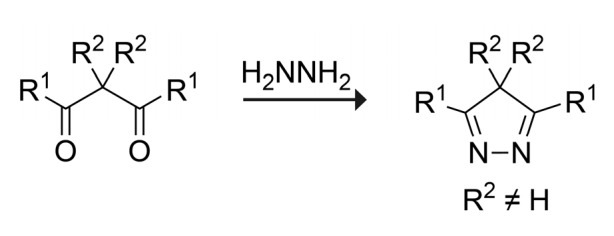
Synthesis of 4*H-*pyrazoles by condensation of 1,3-diketones with hydrazine.

The general method for synthesizing 4*H*-pyrazoles is the condensation reaction of 1,3-diketones with hydrazine, as shown in Scheme 1 [17]. The reaction of hydrazine with diketones containing electron-withdrawing substituents at the 1- and 3-positions predominately leads to the corresponding 5-hydroxy-2-pyrazoline instead of the corresponding 4*H*-pyrazole because the dehydration step becomes difficult. For example, the reaction of 4-nitrobenzoylacetone with hydrazine results in the exclusive formation of the 5-hydroxy-2-pyrazoline [18].

**Scheme 2:**
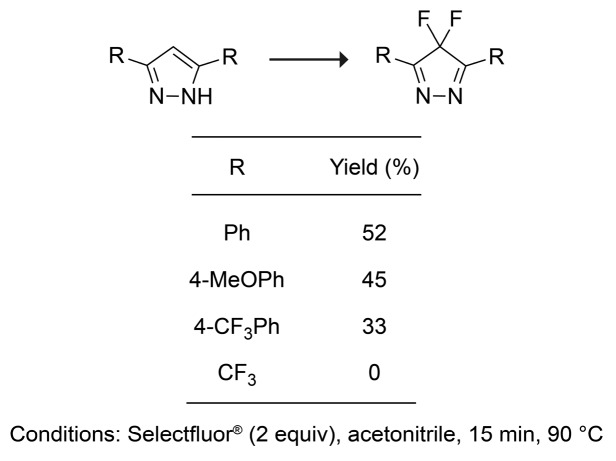
Synthesis of various substituted 4,4-difluoro-4*H*-pyrazoles. Data/Adapted from [19].

The previously reported synthetic route to DFP (R = Ph) and its analogs is shown in Scheme 2 [19,20]. Synthesis is accomplished by reacting a 1*H*-pyrazole with two equivalents of 1-chloromethyl-4-fluoro-1,4-diazoniabicyclo[2.2.2]octane bis(tetrafluoroborate) (Selectfluor^®^), which is a highly reactive electrophilic fluorinating agent [21,22,23]. This route suffers from a limited substrate scope when electron-withdrawing substituents are present at the 3- and 5-positions of the pyrazole ring. The electron-withdrawing substituents lower the nucleophilicity of the fluorinated pyrazole, which makes the addition of a second electrophilic fluorine difficult. To circumvent these shortcomings and develop a generalized method for synthesizing electron-deficient 4*H*-pyrazoles, we sought another route. We also explored the Diels–Alder reactivity of an ensuing 4*H*-pyrazole.

## 2. Results and Discussion

**Scheme 3:**
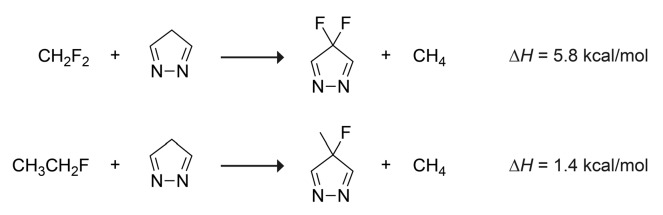
Reaction enthalpies of isodesmic equations to estimate the hyperconjugative antiaromaticity in the 4,4-difluoro-4*H*-pyrazole (top) and 4-fluoro-4-methyl-4*H*-pyrazole (bottom) scaffolds.

To begin, we examined the physical properties of the 4,4-difluoro-4*H*-pyrazole and 4-fluoro-4-methyl-4*H*-pyrazole scaffolds with density functional theory calculations at the M06-2X/6-311++G(d,p)//M06-2X/6-31G(d) level of theory [24]. The rapid Diels–Alder reactivity of the DFP scaffold is attributed to its hyperconjugative antiaromaticity and low-lying LUMO energy [11]. The isodesmic equations in Scheme 3 report on the destabilization that results from hyperconjugative antiaromatic electron delocalization [11,12,13,14,15,25,26]. The reaction enthalpies of the isodesmic equations suggest that hyperconjugative antiaromaticity destabilizes the 4,4-difluoro-4*H*-pyrazole and 4-fluoro-4-methyl-4*H*-pyrazole scaffolds by 5.8 and 1.4 kcal/mol, respectively. The calculated LUMO energies are −2.0 and −1.3 eV for the 4,4-difluoro-4*H*-pyrazole and 4-fluoro-4-methyl-4*H*-pyrazole scaffolds, respectively.

**Figure 1:**
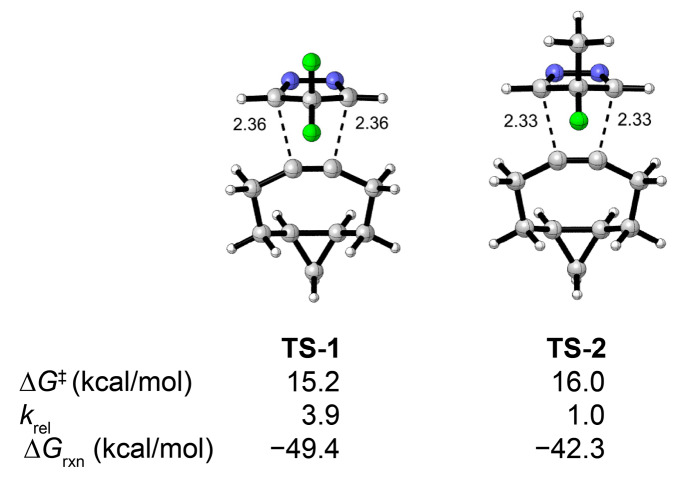
Transition-state geometries and calculated Gibbs free energies of activation (Δ*G*^‡^) and reaction (Δ*G*_rxn_) computed at the M06-2X/6-311++G(d,p)//M06-2X/6-31G(d) level of theory for the Diels–Alder reactions of 4,4-difluoro-4*H*-pyrazole (**TS-1**) and 4-fluoro-4-methyl-4*H*-pyrazole (**TS-2**) scaffolds toward *endo*-bicyclo[6.1.0]non-4-yn-9-ylmethanol (BCN) lacking the 9-hydroxymethyl group. The lengths (Å) of forming bonds are shown. Values of *k*_rel_ were calculated at 298 K with the Arrhenius equation.

Next, we computationally explored the Diels–Alder reactivity of the electron-deficient 4*H*-pyrazole scaffolds. The computed transition-state structures and Gibbs free energies of activation and reaction for the Diels–Alder reactions of the 4-fluoro-4-methyl-4*H*-pyrazole and 4,4-difluoro-4*H*-pyrazole scaffolds towards BCN are shown in Figure 1. Despite the significantly lower LUMO energy and decreased hyperconjugative antiaromaticity of the 4-fluoro-4-methyl-4*H*-pyrazole scaffold relative to the 4,4-difluoro-4*H*-pyrazole scaffold, they have similar Diels–Alder reactivities towards BCN with Gibbs free energies of activation of 16.0 and 15.2 kcal/mol, respectively. The 4,4-difluoro-4*H*-pyrazole scaffold is planar, whereas the 4-fluoro-4-methyl-4*H*-pyrazole scaffold is puckered by 3° into an envelope-like geometry that minimizes the destabilizing effects of hyperconjugative antiaromaticity [15]. The puckering causes the diene to adopt a conformation closer to that of the *syn* Diels–Alder transition state and increases reactivity by reducing the conformational distortion needed to access the transition-state geometry.

**Scheme 4:**
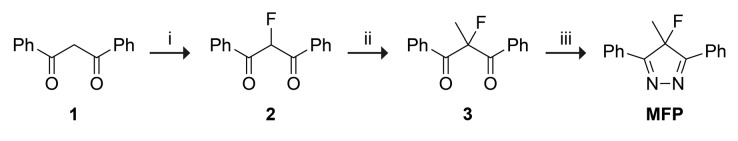
Method A for the preparation of 4-fluoro-4-methyl-3,5-diphenyl-4*H*-pyrazole (MFP). Conditions and yields: i. Selectfluor^®^, acetonitrile, RT, 2.5 h, 96%; ii. MeI, K_2_CO_3_, DMF, 60 °C, 3 h, 88%; iii. H_2_NNH_2_, DCM, reflux, 18 h, 34%.

To test its reactivity experimentally, we set out to synthesize a representative 4-fluoro-4-methyl-4*H*-pyrazole. As the reactivity of 4,4-difluoro-3,5-diphenyl-4*H*-pyrazole (DFP) is known [11], we chose 4-fluoro-4-methyl-3,5-diphenyl-4*H*-pyrazole (MFP) as a target. Initially, we synthesized MFP via the condensation of 2-fluoro-2-methyl-1,3-diphenylpropane-1,3-dione (**3**) with hydrazine (Scheme 4, Method A). The final step in Method A suffered from a low overall yield of 34% because the second dehydration step is difficult when electron-withdrawing substituents, such as a fluoro group, are present [18]. In our hands, this reaction was also inconsistent and ineffective when attempted on a larger scale, leading us to seek a new route.

**Scheme 5:**
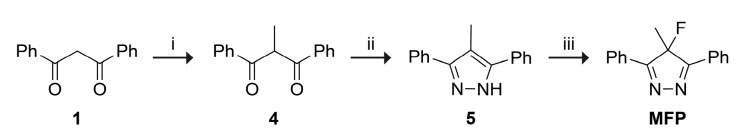
Method B for the preparation of 4-fluoro-4-methyl-3,5-diphenyl-4*H*-pyrazole (MFP). Conditions and yields: i. MeI, K_2_CO_3_, DMF, 60 °C, 30 min, 72%; ii. H_2_NNH_2_, DCM, RT, 18 h, 88%; iii. Selectfluor^®^, acetonitrile, reflux, 1 h, 77%.

Inspired by the successful preparation of 4-chloro-4-methyl-4*H*-pyrazoles by the late-stage chlorination of 1*H*-pyrazoles with *tert*-butyl hypochlorite [7], we turned to late-stage fluorination of a 1*H*-pyrazole. Although this strategy is similar to that for synthesizing DFP [11,19,20], the difficult step in the synthesis of DFP is the low-yielding nucleophilic addition of the second fluorine. That would not be a concern in the preparation of MFP. Fluorination of 1*H*-pyrazoles to yield 4-fluoro-1*H*-pyrazoles has been accomplished previously with Selectfluor^®^ [27], but this method has not been used to prepare 4*H*-pyrazoles. Fluorination of 4-methyl-3,5-diphenyl-1*H*-pyrazole (**5**) with Selectfluor^®^ resulted in a 77% yield of MFP (Scheme 5, Method B). The overall yields were 29% for Method A and 49% for Method B. Moreover, Method B was more consistent in our hands than Method A and proceeded well at larger scales. We anticipated that this new preparatory method will also be useful for accessing derivatives of MFP.

**Figure 2:**
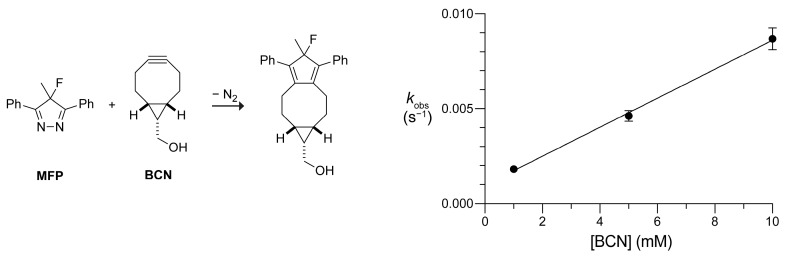
Reaction of *endo*-bicyclo[6.1.0]non-4-yn-9-ylmethanol (BCN) (1, 5, or 10 mM) with 4-fluoro-4-methyl-3,5-diphenyl-4*H*-pyrazole (MFP) (0.1 mM) in 9:1 methanol/water. Values of *k*_obs_ are the mean ± SD for reactions performed in triplicate. The second-order rate constant as calculated from a linear fit of the data was *k* = 0.76 M^−1^s^−1^ (*R*^2^ = 0.99).

With a reliable strategy for the preparation of MFP in hand, we turned our attention to its experimental Diels–Alder reactivity and physiological stability. We measured the rate constant for the Diels–Alder reaction of MFP with BCN in 9:1 methanol/water at ~20 °C under pseudo–first-order conditions. A plot of the observed rate constants with respect to the BCN concentration is shown in Figure 2. From these data, we calculated that the Diels–Alder reaction proceeded with a second-order rate constant of *k* = 0.76 M^−1^s^−1^, which is 7-fold slower than that of DFP with BCN (*k* = 5.2 M^−1^s^−1^) [11].

**Figure 3:**
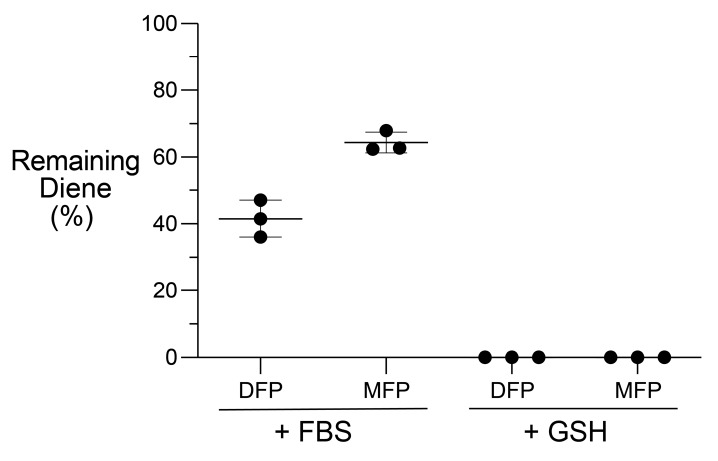
Stability of 44,-difluoro-4-methyl-3,5-diphenyl-4*H*-pyrazole (DFP) and 4-fluoro-4-methyl-3,5-diphenyl-4*H*-pyrazole (MFP) in the presence of fetal bovine serum (FBS) or glutathione (GSH). Percent remaining was determined after an 8 h incubation at 37 °C as measured by UV-vis spectroscopy. In the presence of FBS, (42 ± 5)% and (64 ± 3)% of the DFP and MFP remained intact, respectively. No diene was detectable in the presence of GSH. Data for the stability of DFP in the presence of FBS are from [11].

To assess the physiological stability of MFP and DFP, we incubated both in phosphate-buffered saline (PBS) containing fetal bovine serum (FBS; 10% *v*/*v*) at 37 °C. After 8 h, 42% of the DFP and 64% of the MFP remained intact (Figure 3). Thus, replacement of one fluoro group with a methyl group increased the stability of the 4*H*-pyrazole scaffold in serum by 52%. We also examined the stability of MFP and DFP at 37 °C in PBS containing reduced glutathione (1.0 mM) and oxidized glutathione (0.2 mM), which is a biomimetic redox buffer containing sulfhydryl, amino, and carboxyl groups [28]. We found that neither MFP nor DFP remained intact after an 8 h incubation (Figure 3). The physiological stability studies show that MFP is more stable than DFP, however, both MFP and DFP are compromised in the presence of biological nucleophiles.

## 3. Materials and Methods

### 3.1. Materials

#### 3.1.1. General

All chemicals were from commercial sources and were used without further purification. NMR spectra were acquired with a Avance Neo 400 spectrometer or Avance Neo 500 spectrometer from Bruker (Billerica, MA, USA) and are shown in the Supplementary Materials. Mass spectra were acquired by using positive ionization with an AccuTOF-DART 4G instrument from JEOL (Tokyo, Japan). UV–vis experiments were carried out with an Cary 60 UV–vis spectrometer from Agilent Technologies (Santa Clara, CA, USA) with measurements every 0.1 s for kinetic experiments and absorbance scans for stability studies.

Column chromatography was performed with a Isolera automated purification system from Biotage (Uppsala, Sweden) using prepacked SNAP KP silica gel columns.

The phrase “concentrated under reduced pressure” refers to the removal of solvents and other volatile materials using a rotary evaporator at water aspirator pressure (<20 Torr) while maintaining the water-bath temperature of 40 °C. Residual solvent was removed from samples by the vacuum (<0.1 Torr) achieved by a mechanical belt-drive oil pump.

All procedures were performed in air at ambient temperature (~22 °C) and pressure (1.0 atm) unless indicated otherwise.

#### 3.1.2. Synthesis of 2-Fluoro-1,3-diphenylpropane-1,3-dione (**2**)

1,3-Diphenylpropane-1,3-dione (**1**) (1.5 g, 6.6 mmol) and 1-chloromethyl-4-fluoro-1,4-diazoniabicyclo[2.2.2]octane bis(tetrafluoroborate) (2.34 g, 6.6 mmol) were dissolved in 50 mL of acetonitrile and stirred for 2.5 h. The reaction mixture was concentrated under reduced pressure, and the residue was taken up with DCM. The DCM was washed (3×) with water, dried over Na_2_SO_4_(s), filtered, and concentrated under reduced pressure. The product was purified by chromatography on silica gel, eluting 0–10% *v*/*v* ethyl acetate in hexanes to give 1.5356 g of **2** (96%) as a light orange solid. **^1^H NMR** (400 MHz, CDCl_3_, *δ*): 8.12 (d, *J* = 7.7 Hz, 4H), 7.71–7.56 (m, 2H), 7.51 (t, *J* = 7.6 Hz, 4H), 6.56 (d, *J* = 49.2 Hz, 1H). **^13^C NMR** (101 MHz, CDCl_3_, *δ*): 191.29, 134.54, 129.86, 129.83, 128.81, 97.61, 95.64. **^19^F NMR** (376 MHz, CDCl_3_, *δ*): −186.82 (d, *J* = 49.3 Hz). **HRMS** *m*/*z* calcd for C_15_H_12_FO_2_ [M + H]^+^, 243.08213; found, 243.08385.

#### 3.1.3. Synthesis of 2-Fluoro-2-methyl-1,3-diphenylpropane-1,3-dione (**3**)

Compound **2** (500 mg, 2.06 mmol) and K_2_CO_3_ (427 mg, 2.09 mmol) were dissolved in 5 mL of DMF, and MeI (0.282 mL, 643 mg, 4.53 mmol) was added to the resulting solution. The reaction mixture was heated to 60 °C and stirred for 3 h. The reaction was quenched by the addition of 50 mL of H_2_O, and the reaction mixture was extracted (3×) with ethyl acetate. The combined organic layers were dried over Na_2_SO_4_(s), filtered, and concentrated under reduced pressure. The product was purified by chromatography on silica gel, eluting with 0–20% *v*/*v* ethyl acetate in hexanes to give 462 mg of **3** (88%) as a transparent gel. **^1^H NMR** (500 MHz, CDCl_3_, *δ*): 8.04 (dt, *J* = 8.5, 1.5 Hz, 4H), 7.65–7.56 (m, 2H), 7.47 (t, *J* = 7.8 Hz, 4H), 2.07 (d, *J* = 23.0 Hz, 3H). **^13^C NMR** (126 MHz, CDCl_3_, *δ*): 194.17, 193.98, 133.96, 130.01, 129.97, 128.74, 22.10, 21.92. **^19^F NMR** (471 MHz, CDCl_3_, *δ*): −144.92 (q, *J* = 23.0 Hz). **HRMS** *m*/*z* calcd for C_16_H_14_FO_2_ [M + H]^+^, 257.09778; found, 257.10300.

#### 3.1.4. Synthesis of 4-Fluoro-4-methyl-3,5-diphenyl-4*H*-pyrazole (MFP) Using Method A

Compound **3** (50 mg, 0.206 mmol) was added to an oven-dried flask equipped with a reflux condenser. The flask was purged with N_2_ (g). Hydrazine (6.43 µL, 6.62 mg, 0.206 mL) dissolved in 1 mL of dry DCM was added to the flask. The reaction mixture was heated at reflux with stirring for 18 h and then concentrated under reduced pressure. The product was purified by chromatography on silica gel, eluting with 0–20% *v*/*v* ethyl acetate in hexanes, to yield 17.5 mg **MFP** (34%) as a yellow solid. **^1^H NMR** (400 MHz, CDCl_3_, *δ*): 8.26–8.00 (m, 4H), 7.67–7.44 (m, 6H), 1.90 (d, *J* = 22.8 Hz, 3H). **^13^C NMR** (101 MHz, CDCl_3_, *δ*): 170.48, 132.00, 129.05, 128.10, 128.07, 88.64, 23.97. **^19^F NMR** (376 MHz, CDCl_3_, *δ*): −170.93 (q, *J* = 22.7 Hz). **HRMS** *m*/*z* calcd for C_16_H_14_FN_2_ [M + H]^+^, 253.11410; found, 253.12166.

#### 3.1.5. Synthesis of 2-Methyl-1,3-diphenylpropane-1,3-dione (**4**)

1,3-Diphenylpropane-1,3-dione (**1**) (1.121 g, 5 mmol) and K_2_CO_3_ (1.037 g, 7.5 mmol) were dissolved in 10 mL DMF, and MeI (0.781 g, 5.5 mmol, 0.342 mL) was added to the resulting solution. The reaction mixture was heated to 60 °C and stirred for 30 min. The reaction mixture was diluted with 50 mL of water and extracted with DCM. The organic layer was washed with water, dried over Na_2_SO_4_(s), filtered, and concentrated under reduced pressure. The product was purified by chromatography on silica gel, eluting with 0–10% *v*/*v* ethyl acetate in hexanes, then dried to give 0.8591 g of **2** (72%) as a light pink solid. **^1^H NMR** (500 MHz, CDCl_3_, *δ*): 8.00–7.95 (m, 4H), 7.61–7.57 (m, 2H), 7.48 (t, *J* = 7.8 Hz, 4H), 5.29 (q, *J* = 7.0 Hz, 1H), 1.63 (d, *J* = 7.0 Hz, 3H). **^13^C NMR** (126 MHz, CDCl_3_, *δ*): 197.19, 135.68, 134.34, 133.50, 129.32, 128.91, 128.57, 51.10, 14.39. **HRMS** *m*/*z* calcd for C_16_H_15_O_2_ [M + H]^+^, 239.10720; found, 239.10846.

#### 3.1.6. Synthesis of 4-Methyl-3,5-diphenyl-1H-pyrazole (**5**)

Compound **4** (200 mg, 0.84 mmol) was dissolved in 5 mL of DCM, and hydrazine hydrate (40 µL, 42 mg, 0.84 mmol) was added to the resulting solution. The reaction mixture was stirred for 18 h, which resulted in the solution becoming cloudy. The reaction mixture was then concentrated under reduced pressure and the product was purified by chromatography on silica gel, eluting with 20–50% *v*/*v* ethyl acetate in hexanes to give 173 mg of **5** (88%) as a white solid. **^1^H NMR** (400 MHz, DMSO-*d*_6_, *δ*): 13.07 (s, 1H), 7.64 (d, *J* = 7.6 Hz, 4H), 7.54–7.46 (m, 4H), 7.39 (t, *J* = 7.5 Hz, 2H), 2.28 (s, 3H). **^13^C NMR** (101 MHz, DMSO-*d*_6_, *δ*): 148.19, 129.10, 128.02, 127.99, 127.83, 109.89, 10.60. **HRMS** *m*/*z* calcd for C_16_H_15_N_2_ [M + H]^+^, 235.12352; found, 235.12784.

#### 3.1.7. Synthesis of 4-Fluoro-4-methyl-3,5-diphenyl-4*H*-pyrazole (MFP) Using Method B

Compound **5** (100 mg, 0.43 mmol) and 1-chloromethyl-4-fluoro-1,4-diazoniabicyclo[2.2.2]octane bis(tetrafluoroborate) (151 mg, 0.43 mmol) were added to an oven-dried flask, along with activated 3-Å molecular sieves. The flask was purged with N_2_ (g), and 3 mL of dry acetonitrile was added. The reaction mixture was heated at 90 °C for 1 h, diluted by the addition of 5 mL of ethyl acetate, filtered, and concentrated under reduced pressure. The product was purified by chromatography on silica gel, eluting with 0–10% *v*/*v* ethyl acetate in hexanes to give 84 mg of MFP (77%) as a light yellow solid. Characterization data matched those for MFP prepared according to Method A.

### 3.2. UV–vis Kinetics

Stock solutions of MFP (200 µM) and BCN (20 mM, 10 mM, and 2 mM) were prepared in 9:1 MeOH/H_2_O. A 0.5-mL aliquot of the MFP stock solution was mixed with 0.5 mL of each BCN solution, and the absorbance at 335 nm was monitored until no MFP remained. Each reaction was carried out in triplicate. Values of *k*_obs_ were obtained from the slope of plot of ln(absorbance) versus time. A plot of *k*_obs_ values versus BCN concentration allowed for the calculation of a second-order rate constant.

### 3.3. Compound Stability Studies

#### 3.3.1. Diene Stability in FBS

A solution of MFP (0.1 mM) or DFP (0.1 mM) was prepared in phosphate-buffered saline containing fetal bovine serum (10% *v*/*v*) and dimethyl sulfoxide (DMSO) (2% *v*/*v*). Absorbance was measured at 335 nm (MFP) or 355 nm (DFP) with the baseline corrected to the solution without a diene. The solution was then incubated at 37 °C for 8 h, after which another absorbance reading was taken to determine the amount of remaining diene. Values are the mean ± SD from triplicate assays.

#### 3.3.2. Diene Stability to Glutathione

A solution of MFP (0.1 mM) or DFP (0.1 mM) was prepared in phosphate-buffered saline containing reduced glutathione (1.0 mM), oxidized glutathione (0.2 mM), and DMSO (2% *v*/*v*). Absorbance was measured at 335 nm (MFP) or 355 nm (DFP) with the baseline corrected to the solution without a diene. The solution was then incubated at 37 °C for 8 h, after which another absorbance reading was taken to determine the amount of remaining diene. The assay was performed in triplicate.

### 3.4. Computational Methods

All calculations were performed at the M06-2X/6-311++G(d,p)//M06-2X/6-31G(d) level of theory using Gaussian 16, revision A.03 [29]. Optimized coordinates are listed in the Supplementary Materials.

## 4. Conclusions

We have developed an advantageous synthetic route to fluorinated 4*H*-pyrazoles that employs fluorination of the corresponding 1*H*-pyrazole. This late-stage fluorination strategy could be of utility beyond organic chemistry, given the utility of installing a fluoro group in nucleic acids [30], proteins [31,32], and drugs [33,34,35,36]. In addition, we showed that MFP has potential as a useful click reagent. Replacement of the fluoro group in DFP with a methyl group resulted in only a modest reduction in Diels–Alder reactivity that is accompanied by an increase in physiological stability.

## Supplementary Materials

Supplementary materials can be found at https://www.mdpi.com/1422-0067/21/11/3964/s1 and include M06-2X/6-31G(d) optimized coordinates and ^1^H, ^13^C, and ^19^F NMR spectra.
