# Supplementary material for: Synthesis and Diels–Alder Reactivity of 4-Fluoro-4-Methyl-4H-Pyrazoles
Source: Int J Mol Sci. 2020 May 31;21(11):3964. doi: 10.3390/ijms21113964 (PMC7312747; doi:10.3390/ijms21113964)

Synthesis and Diels–Alder Reactivity of 4-Fluoro-4-Methyl-4*H*-Pyrazoles

Nile S. Abularrage, Brian J. Levandowski and Ronald T. Raines*

Department of Chemistry, Massachusetts Institute of Technology, Cambridge, Massachusetts 02139-4307, United States

* Correspondence: rtraines@mit.edu; Tel.: +1-617-253-1470

Content	Page
Table of Contents	S1
M06-2X/6-31G(d) Optimized Coordinates	S2
NMR Spectra	S8

M06-2X/6-31G(d) Optimized Coordinates

M06-2X/6-311++G(d,p)//M06-2X/6-31G(d)
Gibbs free energies and enthalpies are in Hartree–Fock (HF) units.

4,4-Difluoro-4*H*-pyrazole
G: −424.556174
H: −424.520612

C	-0.02220500	-0.55339400	0.00000000
C	-0.09744800	0.44710800	1.13135100
H	-0.09920100	0.19505700	2.18733200
C	-0.09744800	0.44710800	-1.13135100
H	-0.09920100	0.19505700	-2.18733200
C	1.28506500	-1.33432300	0.00000000
H	2.14112400	-0.65456700	0.00000000
H	1.33148000	-1.96934000	-0.88820400
H	1.33148000	-1.96934000	0.88820400
N	-0.09744800	1.65532900	-0.73095400
N	-0.09744800	1.65532900	0.73095400
F	-1.07213200	-1.44560700	0.00000000

4-Fluoro-4-methyl-4*H*-pyrazole
G: −364.592313
H: −364.555536

C	0.00003000	-0.57384200	0.00000000
C	0.00017100	0.42098000	1.14602000
H	0.00019600	0.15898800	2.19830300
C	0.00017100	0.42098000	-1.14602000
H	0.00019600	0.15898800	-2.19830300
N	0.00017100	1.62220700	-0.73886300
N	0.00017100	1.62220700	0.73886300
F	-1.09013300	-1.36837100	0.00000000
F	1.08957500	-1.36913800	0.00000000

BCN
G: −349.84917

C	0.60913000	0.73533000	-0.27904700
C	-0.62497200	0.75216700	-0.21263700
C	1.88807600	1.44976500	-0.46778900
C	1.60007200	2.94162400	-0.71526700
H	1.03849500	3.04372700	-1.65073800
H	2.55632600	3.45797100	-0.86658400
C	0.85340000	3.60182600	0.42306300

C	-0.65236000	3.62617100	0.53930900
C	-1.58744700	2.99978500	-0.47214000
H	-1.17282400	3.09087600	-1.48247400
H	-2.53603500	3.55126200	-0.47209600
C	-1.88851700	1.51787100	-0.18423700
H	-2.60093700	1.12614400	-0.91921700
H	-2.35817800	1.40099800	0.79932100
H	2.52246300	1.32700100	0.41978300
H	2.43813800	1.01757200	-1.30969600
C	0.10001100	4.88492100	0.20533200
H	1.37022400	3.52153100	1.37826500
H	-1.02044700	3.55866800	1.56176900
H	0.17100800	5.67354700	0.94670600
H	0.02664500	5.23934400	-0.82032700

4,4-Difluoro-4*H*-pyrazole + BCN TS
G: −774.381167

C	-2.09171500	1.12794600	0.46129500
C	0.11795900	0.61367000	-0.17484300
C	0.11795900	-0.61367000	-0.17484300
C	0.80070400	1.91463600	-0.24487100
H	0.36407200	2.55609900	-1.01829900
H	0.69656300	2.44043800	0.71233300
C	0.80070400	-1.91463600	-0.24487300
H	0.36407200	-2.55609900	-1.01830200
H	0.69656300	-2.44043900	0.71233100
C	2.28735800	-1.63430200	-0.55556900
H	2.82471200	-2.58886700	-0.61586200
H	2.35504900	-1.16972600	-1.54595900
C	2.95609900	-0.75701200	0.48375900
C	2.28735800	1.63430300	-0.55556800
H	2.82471200	2.58886800	-0.61586000
H	2.35504800	1.16972800	-1.54595800
C	2.95609900	0.75701200	0.48376000
H	2.92273200	-1.18756700	1.48339000
H	2.92273200	1.18756600	1.48339100
C	4.21397300	0.00000000	0.15754700
H	4.52225300	0.00000000	-0.88551400
C	-2.09171400	-1.12794700	0.46129100
H	5.03554200	0.00000000	0.86570900
N	-1.98131700	-0.69759100	1.68140900
N	-1.98131800	0.69758700	1.68141000
C	-2.55335300	0.00000100	-0.44583100
F	-2.14857700	0.00000300	-1.72569000
H	-2.18151700	-2.18572400	0.23735900

H	-2.18151900	2.18572400	0.23736500
F	-3.91710600	0.00000000	-0.48913000

4-Fluoro-4-methyl-4*H*-pyrazole + BCN TS
G: −714.415951

C	-2.05989700	1.11327300	0.45805500
C	0.13282100	0.61411300	-0.16210400
C	0.13282100	-0.61411300	-0.16210400
C	0.82249500	1.91119700	-0.24001700
H	0.38073800	2.55379400	-1.00992800
H	0.72760100	2.43988000	0.71690100
C	0.82249500	-1.91119700	-0.24001700
H	0.38073800	-2.55379500	-1.00992800
H	0.72760100	-2.43988000	0.71690100
C	2.30604000	-1.63203200	-0.56265700
H	2.84386800	-2.58614700	-0.62950500
H	2.36586000	-1.16500500	-1.55246900
C	2.98366700	-0.75669200	0.47241200
C	2.30604000	1.63203300	-0.56265700
H	2.84386800	2.58614700	-0.62950400
H	2.36586000	1.16500500	-1.55246900
C	2.98366700	0.75669200	0.47241200
H	2.95655700	-1.18778100	1.47205300
H	2.95655700	1.18778100	1.47205300
C	4.23984800	0.00000000	0.13820600
H	4.54154900	0.00000000	-0.90681200
C	-2.05989700	-1.11327400	0.45805500
H	5.06602000	0.00000000	0.84114200
N	-1.94002100	-0.68981500	1.68997200
N	-1.94002100	0.68981500	1.68997200
C	-2.55694000	0.00000000	-0.43154400
F	-2.12097900	0.00000000	-1.73032700
C	-4.09070100	0.00000000	-0.47408200
H	-4.43642300	-0.88708000	-1.01075400
H	-4.50808200	0.00000000	0.53465900
H	-4.43642300	0.88708000	-1.01075400
H	-2.15076500	-2.17446400	0.24399700
H	-2.15076500	2.17446400	0.24399700

4,4-Difluoro-4*H*-pyrazole+BCN Cycloadduct
G: −774.484041

C	-1.65166900	1.09372100	0.28107300
C	-0.23112300	0.67502700	-0.06799000
C	-0.23121800	-0.67515900	-0.06815000

C	0.74066500	1.80940200	-0.24593000
H	0.31660700	2.47410600	-1.01133500
H	0.71027400	2.39396100	0.68652900
C	0.74068700	-1.80939000	-0.24618500
H	0.31678300	-2.47415500	-1.01163600
H	0.71036200	-2.39408200	0.68620400
C	2.19855100	-1.52639100	-0.59329200
H	2.69897800	-2.49174000	-0.73728400
H	2.26257900	-1.00998800	-1.55632600
C	2.92555100	-0.75185200	0.47870300
C	2.19850000	1.52645700	-0.59313900
H	2.69902500	2.49174200	-0.73717000
H	2.26236900	1.01003100	-1.55617500
C	2.92549800	0.75182700	0.47880300
H	2.89635500	-1.22249100	1.45963700
H	2.89626200	1.22234600	1.45979400
C	4.18344400	0.00005000	0.14564500
H	4.49851800	0.00013800	-0.89510700
C	-1.65182700	-1.09384400	0.28077600
H	4.99849100	0.00001600	0.86094700
N	-1.92812200	-0.61754000	1.69597600
N	-1.92802400	0.61714000	1.69611300
C	-2.44192100	0.00006500	-0.45831000
F	-2.30676700	0.00031300	-1.79572000
H	-1.92572600	-2.13851700	0.14426900
H	-1.92529300	2.13850700	0.14475300
F	-3.76583200	0.00007100	-0.20802900

4-Fluoro-4-methyl-4*H*-pyrazole + BCN Cycloadduct
G: −714.508866

C	-1.63282700	1.08461700	0.29192100
C	-0.20952400	0.67343500	-0.07019000
C	-0.20948800	-0.67336600	-0.07025200
C	0.75983600	1.80952000	-0.24349500
H	0.33411100	2.47853600	-1.00444700
H	0.73328300	2.39007700	0.69188400
C	0.75987600	-1.80944600	-0.24349500
H	0.33419500	-2.47853100	-1.00441600
H	0.73326400	-2.38996500	0.69191200
C	2.21680200	-1.52798400	-0.59693800
H	2.71797400	-2.49294000	-0.74206200
H	2.27630000	-1.01183200	-1.56050200
C	2.94822300	-0.75169300	0.47093500
C	2.21670200	1.52790700	-0.59702200
H	2.71797300	2.49278100	-0.74235300

H	2.27602900	1.01158900	-1.56050800
C	2.94815000	0.75170400	0.47090000
H	2.92293700	-1.22150300	1.45244500
H	2.92282000	1.22156100	1.45238700
C	4.20513000	0.00005700	0.13290400
H	4.51590400	0.00004800	-0.90917200
C	-1.63277500	-1.08461400	0.29188700
H	5.02327300	0.00011200	0.84479900
N	-1.87905900	-0.61824200	1.71369700
N	-1.87910700	0.61822800	1.71372600
C	-2.46067800	-0.00002800	-0.43897500
F	-2.22969700	-0.00002100	-1.79413000
H	-1.89605800	-2.13528400	0.16811900
H	-1.89615800	2.13528400	0.16814200
C	-3.96363900	-0.00003400	-0.25138100
H	-4.38412500	0.88523000	-0.73628300
H	-4.38414000	-0.88530300	-0.73627100
H	-4.24788700	-0.00003100	0.80073400

4*H*-Pyrazole
H: −226.047947

C	0.00005000	1.19078100	0.00000000
C	-0.00002000	0.20376600	1.12301000
H	-0.00000500	0.43944500	2.18181400
C	-0.00002000	0.20376600	-1.12301000
H	-0.00000500	0.43944500	-2.18181400
N	-0.00002000	-1.01047800	-0.71851500
N	-0.00002000	-1.01047800	0.71851500
H	0.88463900	1.83887300	0.00000000
H	-0.88440400	1.83904900	0.00000000

Methane
H: −40.44722

C	0.00000000	0.00000000	0.00000000
H	0.63021300	0.63021300	0.63021300
H	-0.63021300	-0.63021300	0.63021300
H	0.63021300	-0.63021300	-0.63021300
H	-0.63021300	0.63021300	-0.63021300

Difluoromethane
H: −238.929204

C	0.00000000	0.00000000	0.49752700
F	0.00000000	-1.09978800	-0.28879200

F	0.00000000	1.09978800	-0.28879200
H	-0.90832400	0.00000000	1.10654300
H	0.90832400	0.00000000	1.10654300

Fluoroethane
H: −178.957045

C	-0.11625000	0.54713700	0.00000000
F	-1.17352100	-0.34294600	0.00000000
H	-0.20587500	1.18162400	0.88901000
H	-0.20587500	1.18162400	-0.88901000
C	1.18768300	-0.21955500	0.00000000
H	2.03814200	0.46839400	0.00000000
H	1.25335200	-0.85531000	0.88665100
H	1.25335200	-0.85531100	-0.88665000

NMR Spectra

^{1}H NMR Spectrum of Compound **2** in $CDCl_3$

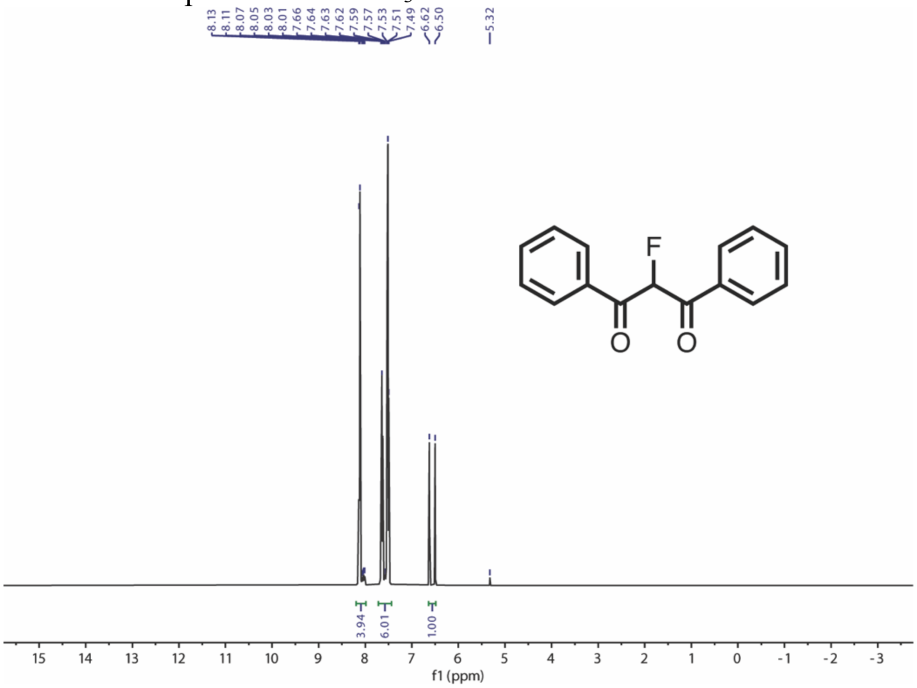

^{13}C NMR Spectrum of Compound **2** in $CDCl_3$

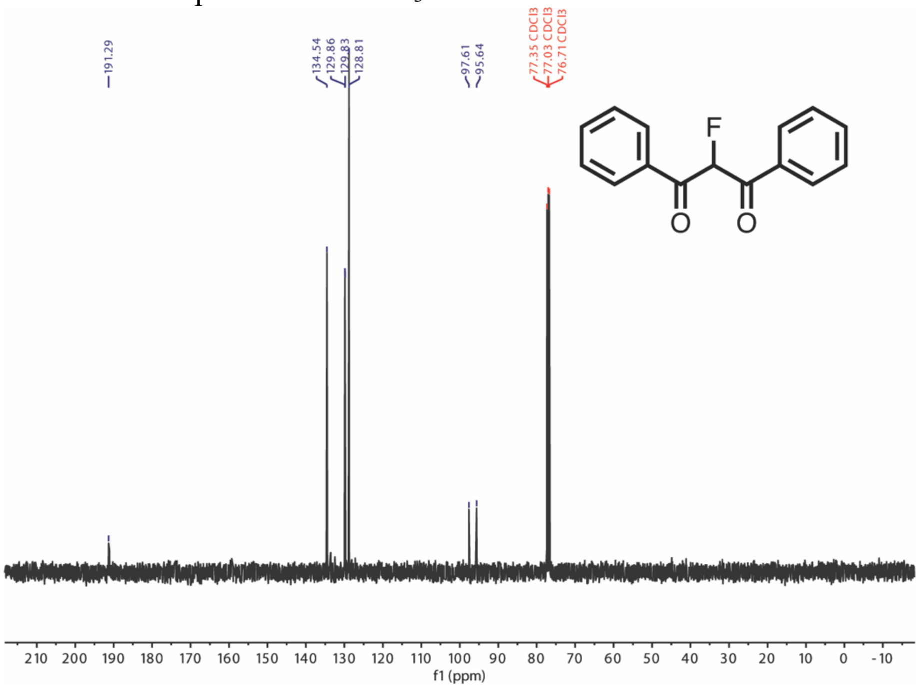

^{19}F NMR Spectrum of Compound **2** in $CDCl_3$

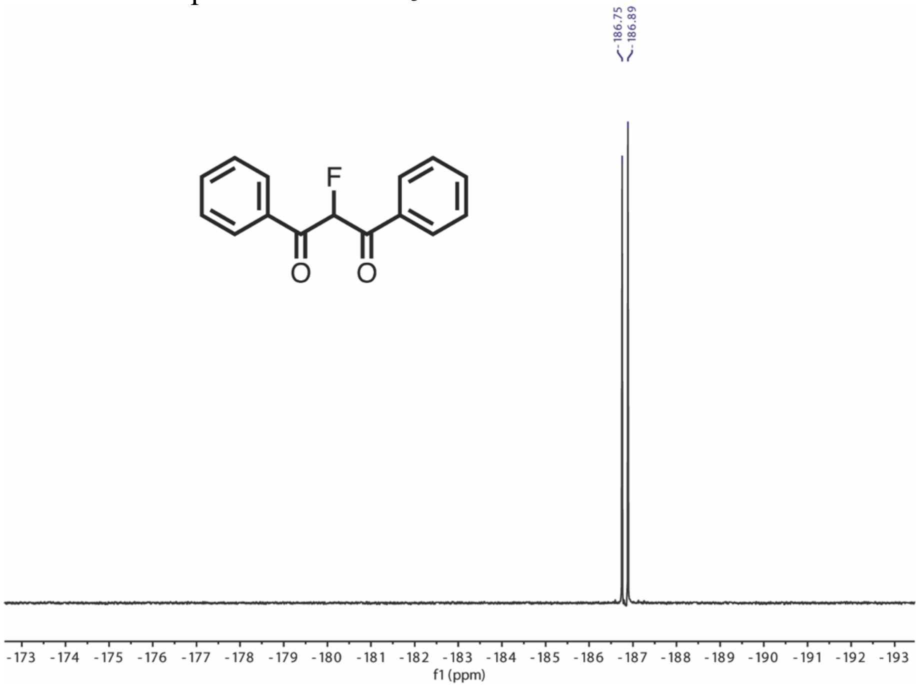

^{1}H NMR Spectrum of Compound **3** in $CDCl_3$

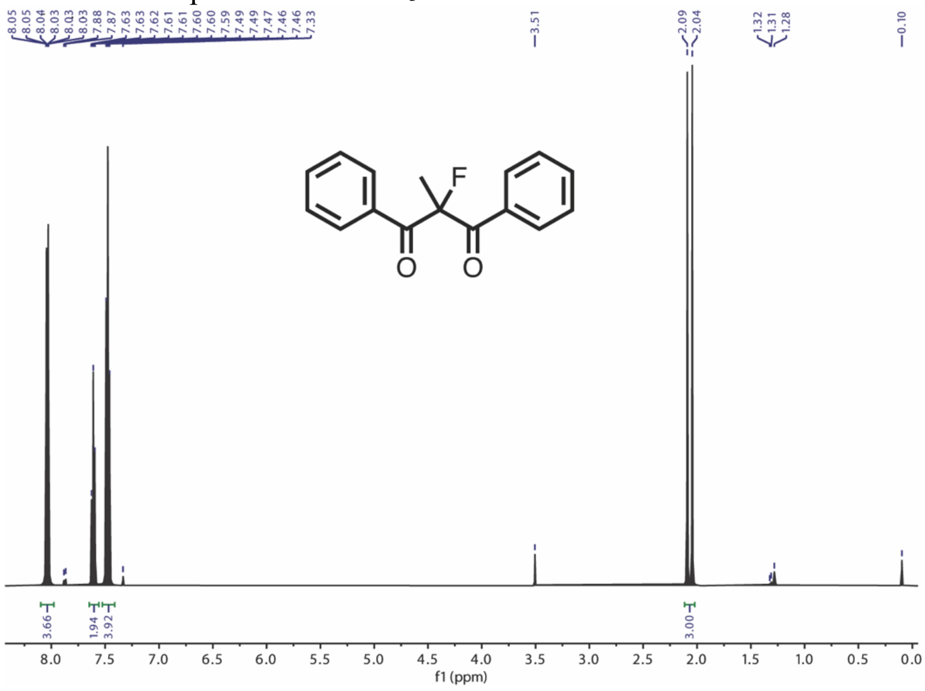

^{13}C NMR Spectrum of Compound **3** in $CDCl_3$

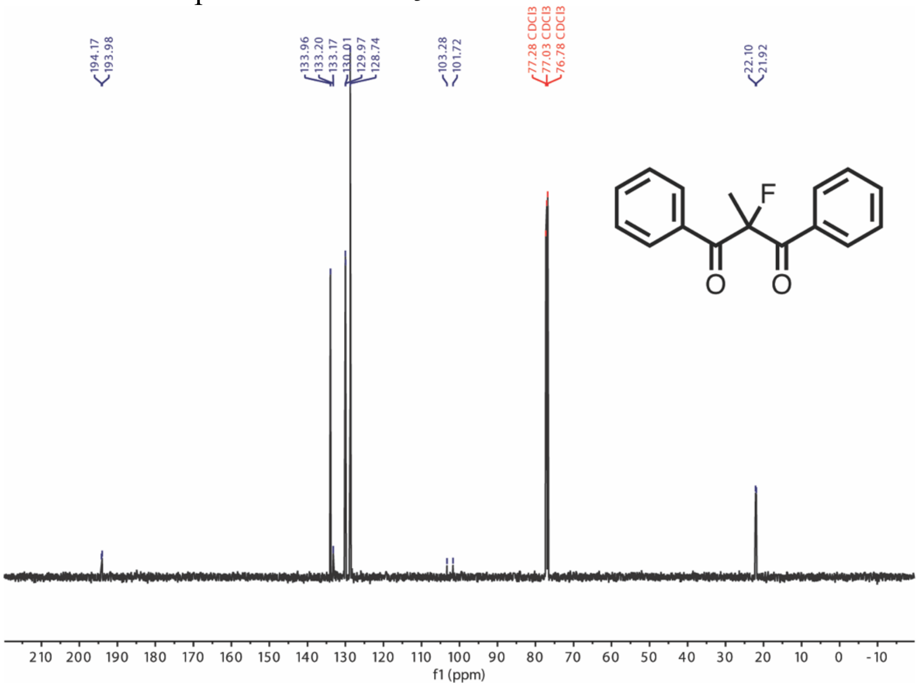

^{19}F NMR Spectrum of Compound **3** in $CDCl_3$

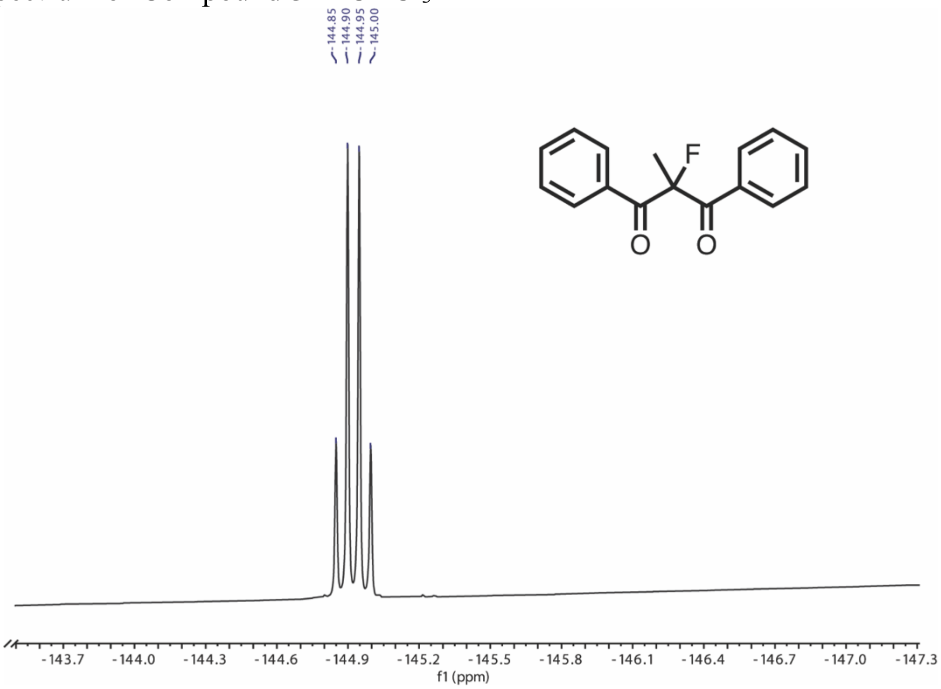

^{1}H NMR Spectrum of Compound **4** in $CDCl_3$

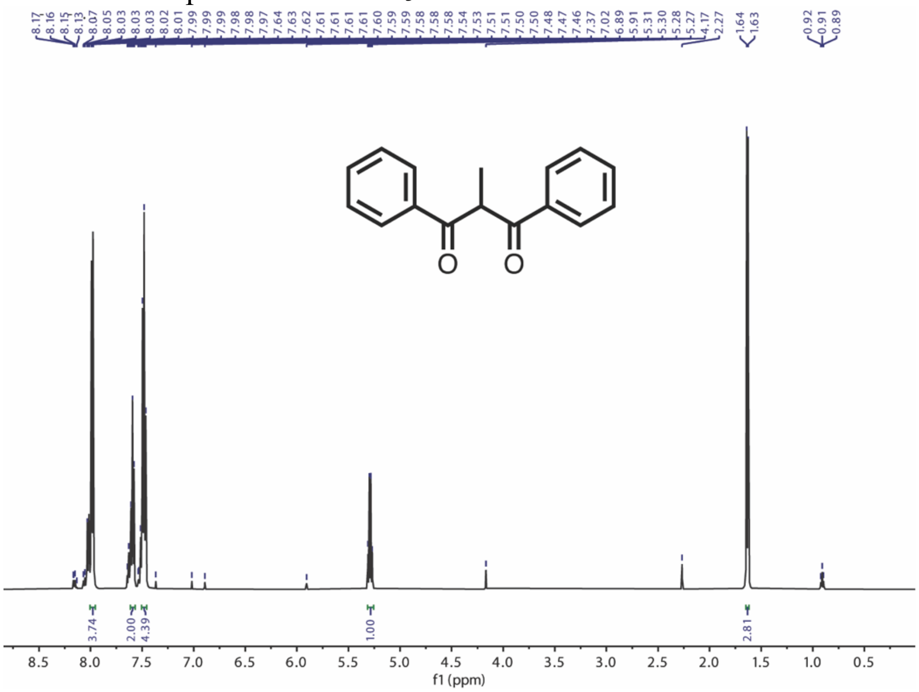

^{13}C NMR Spectrum of Compound **4** in $CDCl_3$

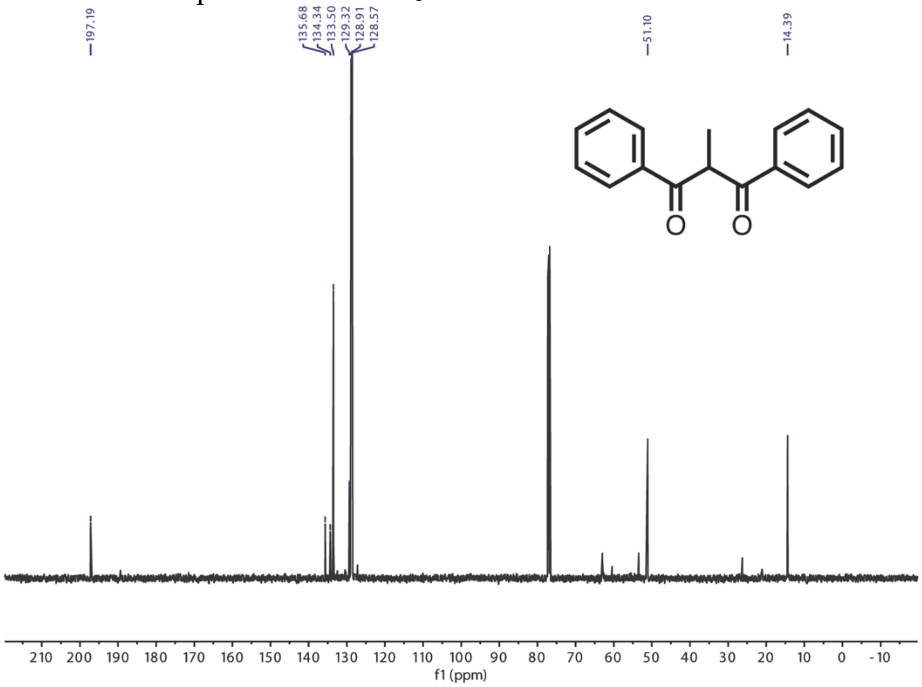

^{1}H NMR Spectrum of Compound **5** in DMSO-d_6

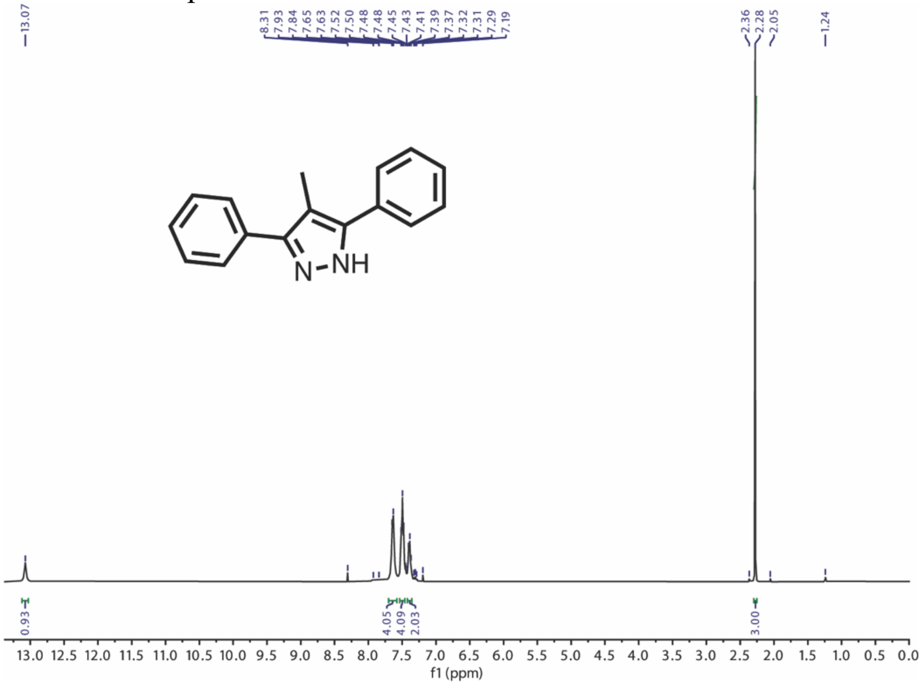

^{13}C NMR Spectrum of Compound **5** in DMSO-d_6

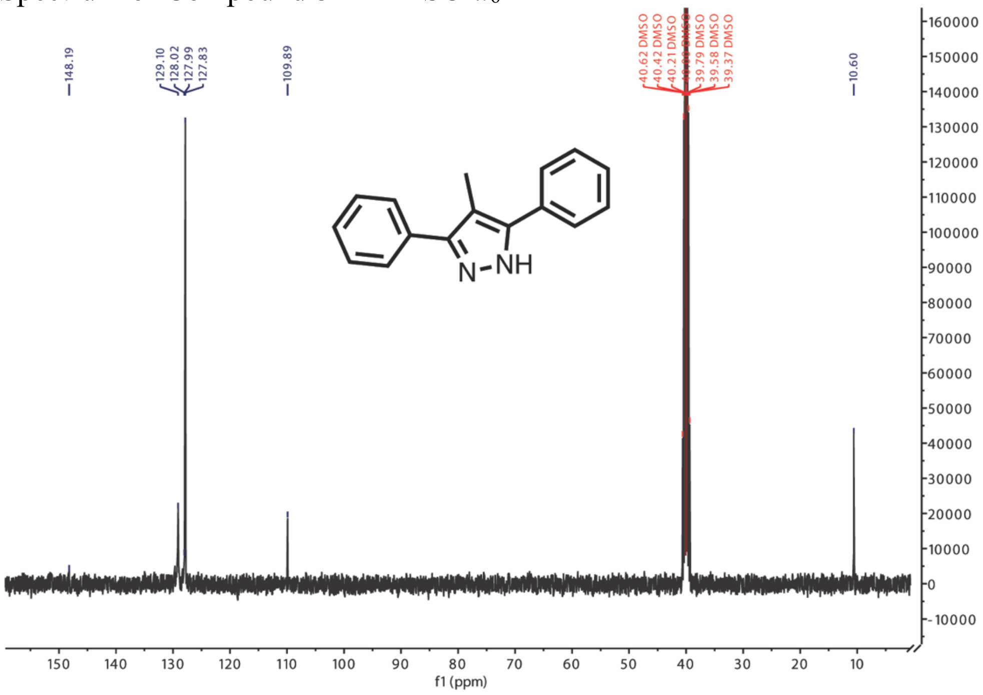

^{1}H NMR Spectrum of **MFP** in $CDCl_3$

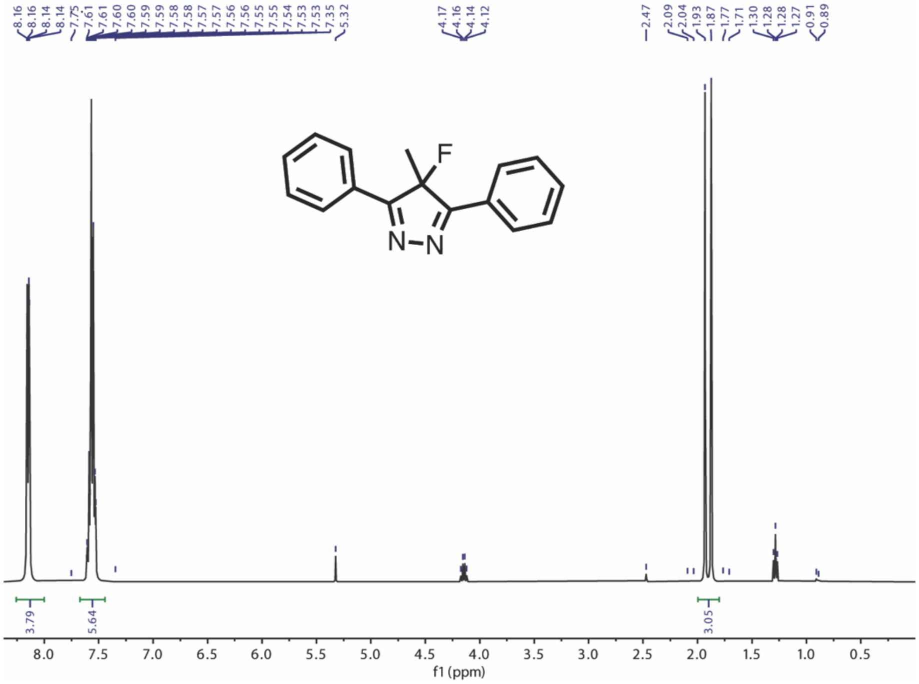

^{13}C NMR Spectrum of **MFP** in $CDCl_3$

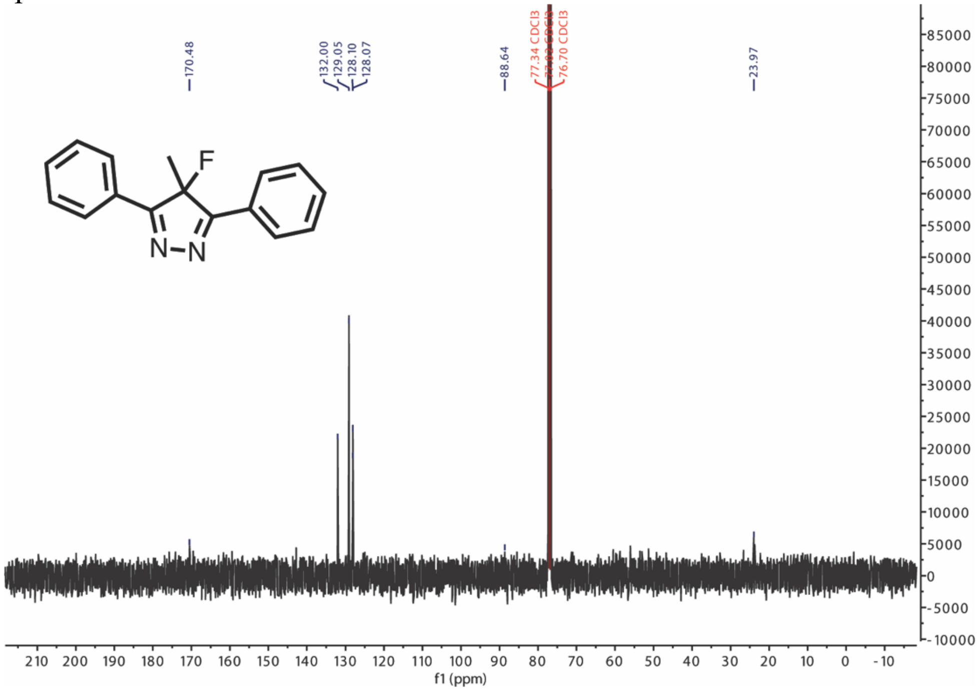

^{19}F NMR Spectrum of **MFP** in $CDCl_3$

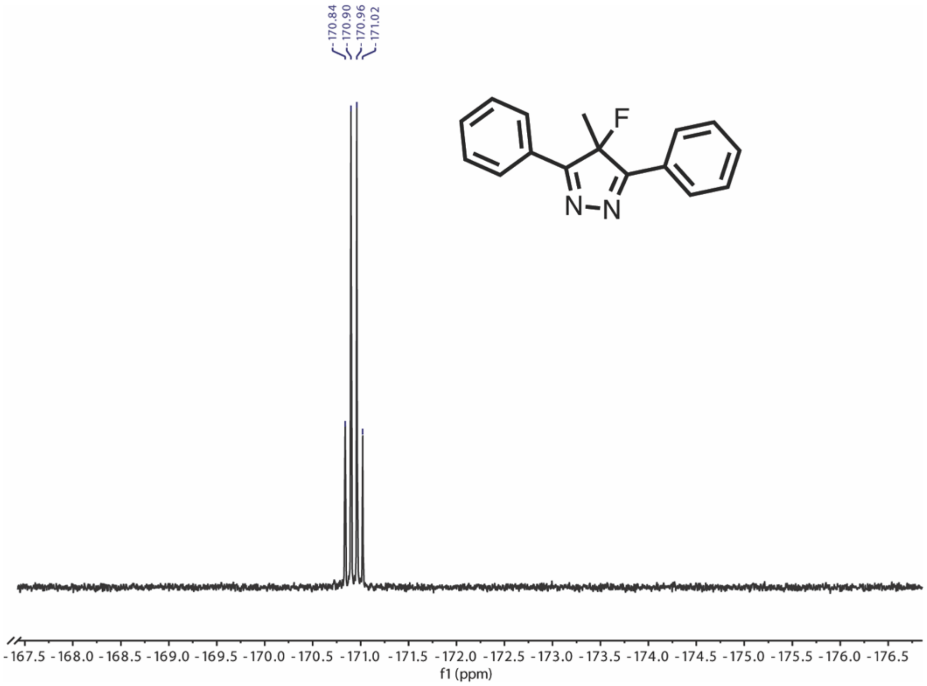

Supplement: Supplementary file 1 [file ijms-21-03964-s001.pdf]
